# Physicians' Perspectives on the Diagnosis and Treatment of Chronic Nonbacterial Osteomyelitis

**DOI:** 10.1155/2017/7694942

**Published:** 2017-01-10

**Authors:** Yongdong Zhao, Fatma Dedeoglu, Polly J. Ferguson, Sivia K. Lapidus, Ronald M. Laxer, Miranda C. Bradford, Suzanne C. Li

**Affiliations:** ^1^Pediatric Rheumatology, Seattle Children's Hospital, Seattle, WA, USA; ^2^Rheumatology, Boston Children's Hospital, Boston, MA, USA; ^3^Department of Pediatrics, University of Iowa Carver College of Medicine, Iowa City, IA, USA; ^4^Pediatric Rheumatology, Goryeb Children's Hospital, Morristown, NJ, USA; ^5^Division of Rheumatology, The Hospital for Sick Children, Toronto, ON, Canada; ^6^Center for Clinical and Translational Research, Seattle Children's Hospital, Seattle, WA, USA; ^7^Pediatrics, Joseph M. Sanzari Children's Hospital, Hackensack University Medical Center, Hackensack, NJ, USA

## Abstract

*Background/Purpose*. Understanding the practices of pediatric rheumatologists in diagnosing and treating chronic nonbacterial osteomyelitis (CNO) can provide important information to guide the development of consensus treatment plans. The objectives of this study were to determine physicians' approaches to (1) diagnosing and monitoring CNO, (2) ordering a bone biopsy, and (3) making treatment decisions.* Methods*. A survey was distributed among members of the Childhood Arthritis and Rheumatology Research Alliance using a web-based questionnaire.* Results*. 121 of 277 (41%) attending physician members completed the survey. Plain radiographs (89%) were most commonly used followed by regional MRI (78%), bone scintigraphy (43%), and whole-body MRI (36%). The top three reasons for performing a biopsy were constitutional findings (66%), unifocal bone lesions (64%), and nocturnal bone pain (45%). Nearly all responders (95%) prescribed nonsteroidal anti-inflammatory drugs (NSAIDs) as initial therapy. For patients who failed NSAID treatment, methotrexate (67%), tumor necrosis factor inhibitors (65%), and bisphosphonates (46%) were the next most commonly used treatments. The presence of a spinal lesion increased the use of bisphosphonate treatment.* Conclusion*. The diagnostic approach and disease activity monitoring for CNO varied among surveyed physicians. Our survey findings provided important background for the development of consensus treatment plans for CNO.

## 1. Introduction

Chronic nonbacterial osteomyelitis (CNO), also known as chronic recurrent multifocal osteomyelitis (CRMO), is an autoinflammatory bone disease of unknown cause that can result in persistent bone pain, bone destruction, functional disability, and pathological fractures. The diagnosis of CNO is based on a history of bone pain, findings of bony tenderness with or without swelling, imaging confirmation of a lytic and/or sclerotic bone lesion, and/or bone edema with negative findings for malignancy and infection of the affected area [1–5]. At diagnosis, bone biopsy is frequently used to exclude infection and malignancy. However, this procedure is not consistently performed across centers [6–9]. Patient characteristics that affect physicians' decisions on whether to request a bone biopsy have not been explored.

Imaging plays an essential role in evaluating children with CNO. Plain radiographs are readily available but not sensitive [10, 11]. Typical findings are osteolytic lesion(s) with surrounding sclerosis and/or hyperostosis. Magnetic resonance imaging (MRI) is very sensitive and can show bone edema, soft tissue inflammationand bony changes such as periosteal reaction, hyperostosis, growth plate damage, or fracture [12–15]. Whole-body MRI imaging is currently considered the imaging modality of choice at the onset of disease [11, 16] but not available in every center. The general pattern of utilization of imaging to monitor disease activity of CNO by pediatric rheumatologists remains unknown.

Due to the rarity of the disease [17] and the lack of randomized controlled trials, treatment remains empirical. Nonsteroidal anti-inflammatory drugs (NSAIDs) are the first-line treatment for most patients with CNO [7, 9]. A prospective study of thirty-seven children showed good response to NSAIDs during the first 3–6 months of illness [9]. Other studies of CNO cohorts have documented the effectiveness of biologic and nonbiologic disease modifying antirheumatic drugs and bisphosphonates in treating CNO not controlled by NSAIDs [7, 8]. Understanding pediatric rheumatologists' practices in diagnosing and treating CNO would inform future refined diagnostic criteria and disease activity monitoring and enable development of standardized treatment regimens (consensus treatment plans) to allow for comparative effectiveness studies.

We conducted a survey of pediatric rheumatologists through Childhood Arthritis and Rheumatology Research Alliance (CARRA). CARRA is a North American organization of more than 425 pediatric rheumatologists, researchers, and research coordinators who are working together to advance the health and quality of life of children living with pediatric rheumatic diseases. Consensus treatment plans for systemic juvenile idiopathic arthritis (JIA) [18], polyarticular JIA [19], proliferative lupus nephritis [20], juvenile localized scleroderma [21], and juvenile dermatomyositis [22] have been developed by CARRA workgroups after initial surveys within CARRA.

The objectives of this study were to determine (1) physicians' approaches in diagnosing and monitoring disease activity, (2) which disease features physicians consider important for ordering a bone biopsy, and (3) physicians' treatment choices.

## 2. Method

This research project was approved by the Seattle Children's Hospital Institution Review Board. The survey was developed based upon feedback from members of the CARRA Scleroderma, Vasculitis, and Rare Diseases (SVRD) subcommittee. Multiple-choice questions were used and case scenarios were presented in addition to general questions. The survey (Appendix  1 in Supplementary Material available online at https://doi.org/10.1155/2017/7694942) was administered through a REDCap database [23]. The survey link was emailed to the 277 attending physicians and 83 trainee physicians via CARRA. Early exit questions were included for physicians without experience in CNO care defined as never directly involved in managing a child with CNO or self-appraisal of insufficient experience. Initial email and two reminders were sent over 4 weeks. Responses were summarized using frequencies and percentages. Reported percentages are out of the full 109 responses except for the minority of questions with incomplete data, for which we reported percentages with counts and partial totals. We compared responses to questions about how long respondents would treat with different medication classes before declaring treatment failure (response options: never/1 month/2 months/3 months/4–6 months) by conducting pairwise *t*-tests of NSAID versus each of four other drug classes: glucocorticoid, disease modifying antirheumatic drug (DMARD), biologic, and bisphosphonate. Tests were adjusted for multiple comparisons using the Bonferroni correction.

## 3. Results

**Figure 1:**
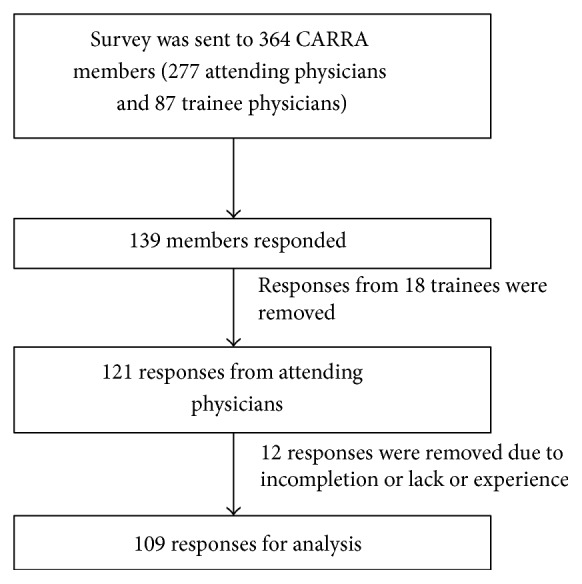
Illustration of responses from CARRA members.

**Figure 2:**
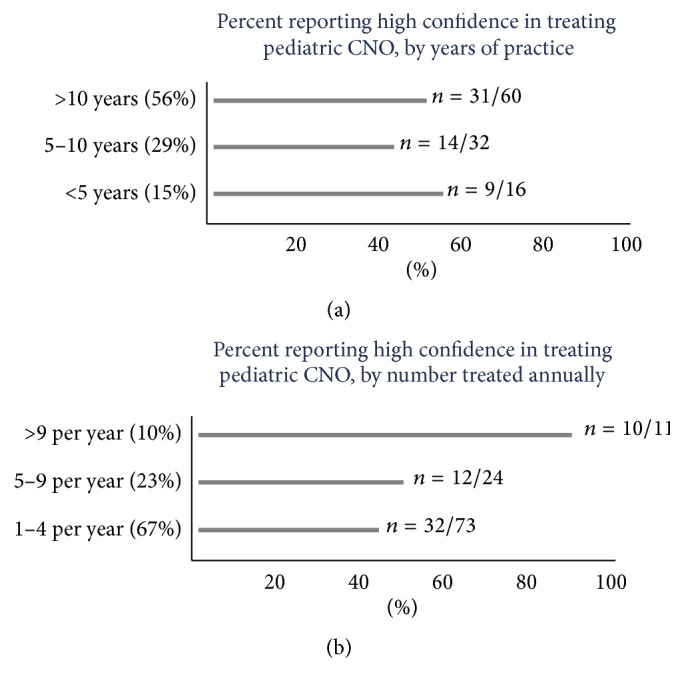
Level of confidence of physicians in treating patients with CNO, (a) by years of practice and (b) by number seen annually.

One hundred and twenty-one of 277 (41%) attending physician members and 18 of 83 trainee physicians from CARRA answered the survey. Only responses from attending physicians who have been involved in care of patients with CNO were retained in the final analytic dataset (*n* = 109) (Figure 1). Sixty-seven percent of responders were currently caring for 1–4 children with CNO and 88% of responders diagnosed 0–3 cases per year. However, 50% (54/108) and 64% of respondents, respectively, felt “completely confident” or “very confident” in treating and diagnosing CNO. The confidence level did not differ by years of experience (Figure 2(a)) but did correlate with the number of CNO patients managed and diagnosed (Figure 2(b)).

Reported frequencies of bone biopsy were never 0%, rarely 12%, sometimes 28%, often 39%, or always 21%. The top three reasons for performing a biopsy were constitutional changes such as fever, weight loss, night sweats (66%), unifocal bone lesion (64%), and nocturnal bone pain (45%). The top three reasons for not performing a biopsy were involvement of “typical sites” (64%), the presence of multiple bone lesions (61%), and CNO-associated conditions such as psoriasis, inflammatory bowel disease (IBD), or enthesitis-related arthritis (ERA) (50%). “Typical sites” were identified as the clavicle (54%), metaphysis of long bones (36%), vertebral bodies (28%), long bones in lower extremity (27%), mandible (21%), long bones in upper extremity (15%), epiphysis of long bones (6%), diaphysis of long bones (5%), other (7%) including pelvis, and sternum. The reasons for obtaining or not obtaining a bone biopsy were similar regardless of whether the physician reported ordering biopsies rarely or often (data not shown).

The top three ways of identifying bone biopsy site were ease of access (65%), leaving the decision to the orthopedic surgeon/interventional radiologist (64%), and the presence of a lytic lesion on X-ray (30%).

The following histologic features were considered to be indicative of CNO: presence of plasma cells, macrophages, and neutrophils (69%), reparative changes such as fibrosis (51%), normal bone (20%), signs of necrosis (19%), unclear reason (15%), biopsy rarely obtained (12%), and other (6%).

**Figure 3:**
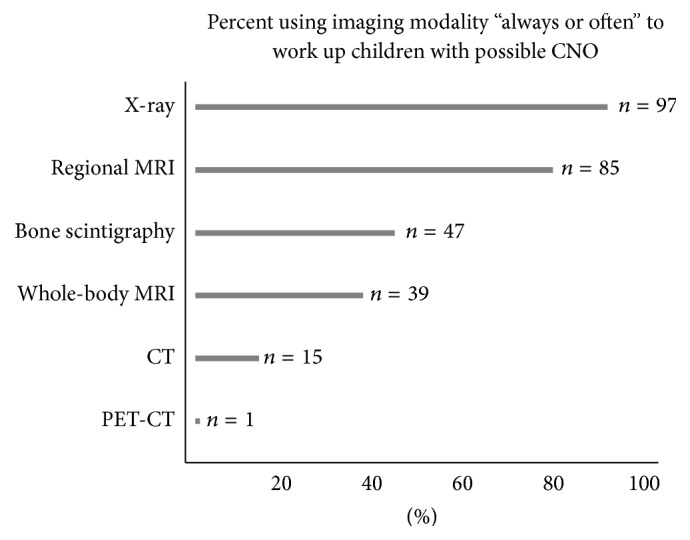
Imaging choices for suspected CNO.

Among all imaging modalities used often or always, X-rays (89%) were most commonly used diagnostic imaging modality, followed by regional MRI (78%), and bone scintigraphy (43%) (Figure 3). Fifty-one percent of responders used imaging regularly to monitor disease activity. Of these 62 responders, 54% monitored the disease activity every 6 months and 25% obtained imaging every 12 months. Thirty-four percent of responders only used imaging when new symptoms occurred and 14% of responders did not routinely use imaging to monitor disease activity.

Among responders who used MRI for disease monitoring (*n* = 39), 59% found the short tau inversion recovery (STIR)/fat suppressed sequences helpful, 38% found the T2 and contrast sequences helpful, and 26% found the T1 sequence helpful. The top 3 MRI findings thought indicative of active diseases were bone edema (43%), periosteal reaction (37%), and soft tissue inflammation (28%). The top 3 MRI findings most concerning for poor prognosis were vertebral compression (30%), fracture (19%), and physeal irregularity (13%).

Physicians defined features that indicate active disease as new lesions identified from imaging (92%), elevated ESR and/or CRP (91%), pain localized to known sites (86%), focal bone swelling and/or warmth (83%), focal tenderness at known sites without allodynia (82%), active arthritis (53%), fever (45%), and functional limitation of joints/limbs (24%).

Features of inactive disease were defined by physicians as resolution of constitutional symptoms (91%), absent focal tenderness and/or warmth and/or swelling of known CNO lesions (85%), normal erythrocyte sedimentation rate (ESR), c-reactive protein (CRP) (83%), complete resolution of pain at known CNO lesions (71%), resolution of synovitis if present with active disease (71%), absence of inflammation confirmed by imaging (57%), no new CNO lesions confirmed by whole-body MRI (49%), and normal function of the affected sites (37%).

Medications that were often or always used by a high proportion of responders included nonsteroidal anti-inflammatory drugs (NSAIDs) (naproxen, 80%; indomethacin 41%; celecoxib, 12%; other NSAIDs, 19%). Medications often or always used by a lesser proportion of responders include methotrexate (34%), adalimumab or infliximab (26%), etanercept (17%), glucocorticoids (13%), bisphosphonate (13%), sulfasalazine (4%), other DMARDs (2%), and azithromycin (<1%). These proportions were similar across categories of years of experience, number of patients diagnosed, or number of patients treated (data not shown).

**Figure 4:**
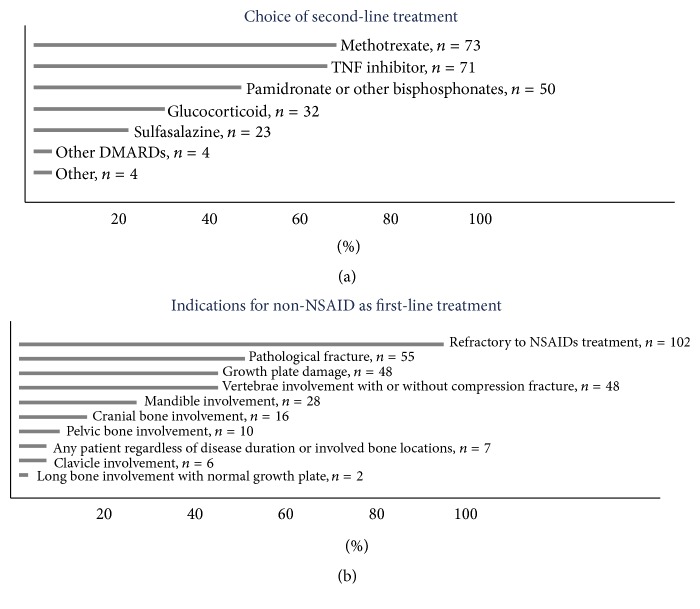
(a) Frequency of use for non-NSAID as second-line treatment; (b) clinical features indicating non-NSAID as first-line treatment.

Almost all responders (95%) routinely prescribed an NSAID as initial therapy. For patients who failed NSAID treatment, methotrexate (67%), TNF-*α* inhibitors (65%), and bisphosphonates (46%) were the next most commonly used treatments (Figure 4(a)). The top 5 reasons to advance therapy with nonbiologic DMARDs and/or biologics and/or bisphosphonate were disease refractory to NSAID treatment (94%), pathological fracture (51%), vertebral involvement with or without compression fracture (44%), growth plate damage (44%), and mandible involvement (26%) (Figure 4(b)). After patients achieved inactive disease defined by the treating physician, the most commonly reported duration for further treatment was 4–6 months (25%) and 7–12 months (28%), or the decision depended on initial severity and/or location of affected sites (30%).

Seventy-eight percent of responders have used glucocorticoids to treat CNO. Physicians tended to treat with steroids for short durations: 1–3 weeks (51%, 43/85) and 4–6 weeks (36%, 31/85). The most common dosage ranges included 0.25–0.5 mg/kg/day (22%, 17/78), 1 mg/kg/day (51%, 40/78), or 1-2 mg/kg/day (21%, 16/78). The maximum daily dose ranged between 10 and 80 mg with a median of 60 mg.

The minimum trial period of treatment needed before declaring failure varied across medication classes, with physicians reporting consideration of treatment failure after 2-3 months of use of NSAIDs (63%), nonbiologic DMARDs (77%), or biologic DMARDs (78%) when active disease persisted. However, as early as 1 month of use of glucocorticoids (64%) and as late as 3–6 months of use of bisphosphonates (61%) were regarded as treatment failures. Pairwise comparisons between NSAIDs and other drug classes found that, on average, physicians reported 1-month shorter trial periods for glucocorticoid (*p* < 0.01) and 1-month longer trial periods for DMARD, biologic, and bisphosphonate (*p* < 0.01) compared to NSAIDs.

Three hypothetical cases were presented. Case  1 had a unifocal lesion in an upper extremity with mildly increased ESR and CRP. Case  2 had vertebral lesions and case  3 had multiple pelvic lesions. The top three leading choices of treatment for all cases were NSAIDs, followed by DMARDs and TNF-*α* inhibitors (case  1: endorsed by 78%, 21%, and 10% of respondents, resp.; case  2: 60%, 29%, and 27%; case  3: 52%, 50%, and 31%). The presence of a spinal lesion increased the use of bisphosphonate treatment (26% for case  2 versus 4–6% for cases  1 and 3). Pamidronate (79%, 22/28) was the most commonly used bisphosphonate followed by zoledronic acid (21%, 6/28) in case  2. The most commonly used dosing of pamidronate was 1 mg/kg/dose monthly or daily for 3 days and repeat every 3 months (maximum dose 60 mg). The first-time dose was often reduced to 0.5 mg/kg in two daily doses for better tolerance. The dosing of zoledronic acid was reported as 0.0125 mg/kg/dose (maximum dose of 4 mg) and repeated every 6 months.

Physicians reported continuing treatment for cases  1–3 for an additional 4–6 months (39%, 24%, and 27%) or 7–12 months (31%, 39%, and 39%) after these patients achieved inactive disease.

**Figure 5:**
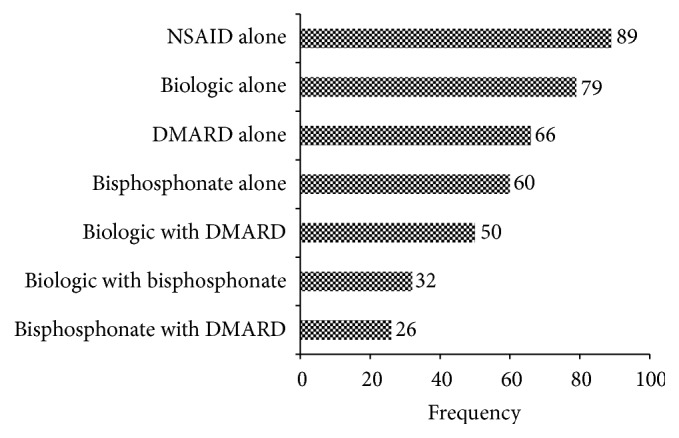
Preferred treatment choices for future comparative effectiveness trials in CNO.

Fifty-eight percent of responders answered “yes” to being interested in participating in a comparative effectiveness study of different treatments for CNO. Among all responders, the preferred treatment choices for future comparative effectiveness studies in CNO were NSAIDs alone (89%), biologics only (79%), nonbiologic DMARDs only (66%), bisphosphonates (60%), biologics with nonbiologic DMARDs (50%), biologics with bisphosphonates (32%), and bisphosphonates with DMARD (26%) (Figure 5).

## 4. Discussion

To our knowledge, this is the first study to assess the practice patterns of pediatric rheumatologists in diagnosing and managing CNO. Using a CARRA-wide survey, we captured the important aspects of disease management including clinical features to obtain bone biopsy, selection of imaging modality, frequency of imaging to monitor disease activity, treatment choices, and treatment duration.

Those physicians with more clinical experience in diagnosing and managing CNO were more confident in their abilities caring for these patients than those with less clinical experience caring for patients with CNO. However, the years of practice did not affect the confidence level.

Bone biopsy is frequently performed to exclude infection and malignancy but was not considered essential to confirm a diagnosis of CNO by at least 40% of physicians. Interestingly, these physicians used similar criteria to decide which cases to biopsy regardless of reported frequency of ordering bone biopsy.

Constitutional changes, unifocal bone lesion, and nocturnal bone pain raise concerns of infection and/or malignancy. Thus, children with these features are often referred for bone biopsy. Although these symptoms are not specific and may be present in some children with CNO, most physicians request a bone biopsy to exclude other causes. Conversely, when multifocal bone lesions exist, typical sites are involved, or associated conditions such as psoriasis, IBD, and ERA are present, physicians may deem bone biopsy unnecessary. Whether ERA is associated with CNO remains controversial. In a pediatric CNO cohort, inflammatory arthritis occurred in up to 80% of children initially or during the course of the disease and five of 30 patients (17%) satisfied the European Spondyloarthropathy Study group criteria for spondyloarthropathy [5]. Our questionnaire did not separate the three associated diseases so it is not possible to compare the responses pertaining to individual condition. Biopsy sites were mostly determined by ease of access and by the proceduralists' preference. Greater than 20% of responders regarded clavicle, metaphysis of long bones, vertebral bodies, long bones in lower extremity, and mandible as typical sites.

Jansson et al. have developed a scoring system in an attempt to determine the threshold of obtaining a bone biopsy in a child with suspected osteomyelitis [24]. CNO was associated with a normal blood cell count (13 points), symmetric bone lesions (10 points), lesions with marginal sclerosis (10 points), normal body temperature (9 points), a vertebral, clavicular, or sternal location of lesions (8 points), presence of >1 radiologically proven lesion (7 points), and C-reactive protein level >1 mg/dL (6 points). A score ≥39 has a positive predictive value of 97% for CNO. The authors suggested obtaining bone biopsy only in bone-scintigraphy negative cases or unifocal lesion with a score ≤28 and clinical monitoring for children with a score of 29–38.

In suspected cases, whole-body MRI has the highest sensitivity to detect active bone lesions [11, 25]. However, only 36% of participants often or always use this technique. Bone scintigraphy is often or always used more than whole-body MRI, which may be due to its better availability. However, the lack of sensitivity [26, 27] and the radiation exposure limit bone scan for further use as a disease-monitoring tool. There is therefore a need to raise awareness of the superiority of whole-body MRI and increase access to this technique for affected children.

Only a limited number of participants used MRI to monitor disease activity. Characteristics such as bone edema, periosteal reaction, and soft tissue inflammation were identified as indicative of active disease which have been shown to be reversible after treatment in various studies [9, 15]. Characteristics such as vertebral compression, fracture, and physeal irregularity were deemed as indicators for poor prognosis and may require more aggressive treatment as suggested by other studies [28, 29].

Treatment options vary for individual patients. Most physicians prescribed NSAIDs as first-line treatment which is in line with published studies [6, 7, 9]. However, when vertebral involvement, pathological fractures, or growth plate damage was present, treatment escalation with DMARDs and/or biologics and/or bisphosphonates was considered appropriate, and there was variability in the choice of these second-line agents. These responses highlighted the recognition of critical lesion sites and were consistent with the responses on poor prognostic factors from MRI findings.

In children with CNO who failed NSAIDs, there has not been a consensus treatment plan among pediatric rheumatologists. Our CARRA survey results suggested that nonbiologic DMARDs, TNF inhibitors, and bisphosphonates were most commonly used by the treating physicians and need further study which could be done using comparative effectiveness research.

Vertebral lesions were associated with higher risk of compression fracture. In this setting, bisphosphonates have been reported to be effective in reducing the pain and inflammation in bone [29–33]. Most surveyed physicians used dosing similar to previous reports.

The definition of active and inactive diseases has not been clearly described due to the lack of specific disease markers. Beck et al. [9] developed a PedCNO score to assess response to treatment. The PedCNO score includes ESR, number of radiological lesions, and severity of disease estimated by the physician, severity of disease estimated by the patient or parent, and the childhood health assessment questionnaire (CHAQ). In this prospective study, 54% of patients treated with NSAID achieved PedCNO 70 (at least 70% improvement in at least three out of five core set variables, with no more than one of the remaining variables deteriorating by more than 70%) at 12 months. In our study, the majority of physicians agreed to define features of active disease as new lesions identified from imaging (92%), elevated ESR and/or CRP (91%), pain localized to known sites (86%), focal bone swelling and/or warmth (83%), focal tenderness at known sites without allodynia (82%), and active arthritis (53%). These results are consistent with other studies. Presence of arthritis has been reported in CNO [3, 6, 34] and deemed as active disease by majority of responders. Features of inactive disease were defined by physicians as the resolution of the above in addition to resolution of constitutional symptoms (91%) and resolution of abnormal MRI signals (68%). Likely a composite of pain complaint, physical, laboratory, and imaging findings is necessary for a comprehensive determination of disease status.

Our study has several limitations. Firstly, the response rate from pediatric rheumatologists was low; hence there could have been survey-sampling bias. Secondly, there may be deviations between answers to the survey and actual practice. Thirdly, the definition of CNO may vary among physicians.

Despite the limitations of our study, we were able to identify key issues such as diagnostic approaches, disease monitoring, and treatment selections after failing NSAIDs as well as definition of disease activity. Results from this study will guide further discussion within a focused group to develop consensus treatment plans for children with CNO.

Our results showed that majority of pediatric rheumatologists manage fewer than five patients with CNO per year. Similar to the prevalence of other pediatric rheumatic diseases such as systemic lupus erythematosus and juvenile dermatomyositis, CNO is uncommon and will need a collaborative effort across centers for future prospective clinical studies.

## 5. Conclusion

The diagnostic approach and disease activity monitoring for CNO varied among physicians. NSAIDs remained the first-line treatment for CNO. Methotrexate, TNF inhibitors, and bisphosphonates were most commonly used after NSAIDs failed. These findings provided important background to move forward with development of consensus treatment plans for CNO.

## Supplementary Material

An online survey through Redcaps were used to collect information from CARRA members regarding their practicing patterns of diagnosing and managing children with CNO.
