# Supplementary material for: Physicians' Perspectives on the Diagnosis and Treatment of Chronic Nonbacterial Osteomyelitis
Source: Int J Rheumatol. 2017 Jan 10;2017:7694942. doi: 10.1155/2017/7694942 (PMC5259599; doi:10.1155/2017/7694942)
Supplement: Supplementary file 1 — An online survey through Redcaps were used to collect information from CARRA members regarding their practicing patterns of diagnosing and managing children with CNO. [file 7694942.f1.pdf]

Physician perspective of diagnosis and treatment of CRMO/CNO

Dear CARRA Member:

Our CRMO/CNO (chronic recurrent multifocal osteomyelitis, or chronic nonbacterial osteomyelitis) group is interested in improving care of CNO patients. We need your help to better understand the prevalence, current diagnostic and treatment strategies of CNO. This survey is intended for all CARRA members; if you have never treated or are not currently treating CNO patients, you will only have to answer 3 questions (early opt out). The full survey will take approximately 10-15 minutes to complete. We appreciate your help and welcome your feedback. If you are interested in helping more with this project, please contact Dan Zhao, yongdong.zhao@seattlechildrens.org.

Sincerely,

Dan Zhao,
Fatma Dedeoglu,
Polly Ferguson,
Suzanne Li,
Ron Laxer,
Sivia Lapidus

1.Please indicate which of the following you are.	◯ Pediatric (or med/peds) rheumatology attending<br>◯ Pediatric (or med/peds) rheumatology fellow<br>◯ Pediatric rheumatology nurse pratitioner<br>◯ Other health care related person: if selected, this will opt you out of this survey
2. How many years have you practiced pediatric rheumatology (including fellowship training)?	◯ < 5<br>◯ 5-10<br>◯ >10
3. Have you ever been involved in the care of children with CNO? If you select no, then after clicking you will be directed to the end of the survey, thank you for your help.	☐ Yes<br>☐ No
4.How many children with CNO do you personally take care of on a regular basis each year?	◯ 1-4<br>◯ 5-9<br>◯ >=10
5.How many patients do you personally diagnose with CNO each year?	◯ 0-3<br>◯ 4-6<br>◯ >6
6.How confident are you with diagnosing children with CNO?	◯ Completely confident<br>◯ Very confident<br>◯ Moderately confident<br>◯ Somewhat confident<br>◯ Not at all confident
7.How confident are you with treating children with CNO?	◯ Completely confident<br>◯ Very confident<br>◯ Moderately confident<br>◯ Somewhat confident<br>◯ Not at all confident

8.How often do you obtain a bone biopsy when evaluating a new patient with possible CNO?

◯ Never
◯ Rarely (< 25%)
◯ Sometimes (25-50%)
◯ Often (51-90%)
◯ Always (>90%)

9.What factors make you decide to obtain a bone biopsy? CHOOSE ALL THAT APPLY.

☐ Unifocal bone lesion
☐ Diaphyseal lesion
☐ Cranial lesion
☐ Constitutional changes (i.e. fever, weight loss, night sweats)
☐ Elevated ESR/CRP
☐ Nocturnal bone pain
☐ Persistent bone pain after 3 months of NSAID treatment
☐ Absence of associated conditions, i.e. psoriasis, IBD, enthesitis-related arthritis
☐ Absence of family history of psoriasis, IBD, ankylosing spondylitis
☐ Other, please specify

____

10.What factors would make you think a bone biopsy is NOT necessary? CHOOSE ALL THAT APPLY.

☐ Multiple bone lesion
☐ Typical sites
☐ Absence of constitutional changes (i.e. weight loss, fevers)
☐ Normal or mildly elevated ESR/CRP
☐ Absence of nocturnal bone pain
☐ Presence of associated conditions, i.e. psoriasis, IBD, enthesitis-related arthritis
☐ Presence of family history of psoriasis, IBD, ankylosing spondylitis
☐ Well appearance
☐ Longer duration of disease between 3-6 months
☐ Longer duration of disease between 6-12 months
☐ Longer duration of disease > 12 months
☐ Other,

____

11.What do you consider as typical sites enough NOT to obtain a bone biopsy? CHOOSE ALL THAT APPLY.

☐ Metaphysis of long bones
☐ Epiphysis of long bones
☐ Diaphysis of long bones
☐ Vertebrae
☐ Mandible
☐ Clavicle
☐ Long bones in lower extremity
☐ Long bones in upper extremity
☐ Other

____

12.If there are multiple sites of bone disease, how do you choose which site(s) to biopsy? CHOOSE ALL THAT APPLY.

☐ Leave the decision to the orthopedic surgeon or intervention radiologist
☐ Lytic lesion on X ray
☐ long bone
☐ vertebrae
☐ clavicle
☐ pelvic bone
☐ Accessible for biopsy
☐ other

Please specify. ______________________________

13.What findings from bone pathology are commonly seen in your patients with CNO? CHOOSE ALL THAT APPLY.

- ☐ Normal bone
- ☐ Reparative changes such as fibrosis
- ☐ Presence of plasma cells, macrophages, and neutrophils
- ☐ Signs of necrosis
- ☐ Biopsy rarely obtained
- ☐ Don't know
- ☐ Other

Please specify. ______________________

14.How many cases have you diagnosed initially as CNO but later transformed to malignancy?

- ○ Never
- ○ 1
- ○ >=2

15.How often do you use the following imaging modalities to work up children with possible CNO in your institution?

	Never	Rarely	Sometimes	Often	Always
X ray	○	○	○	○	○
CT	○	○	○	○	○
Bone scan	○	○	○	○	○
PET-CT	○	○	○	○	○
Regional/focused MRI	○	○	○	○	○
Whole body MRI	○	○	○	○	○
Other	○	○	○	○	○

Please specify. ______________________

16. Do you routinely obtain imaging to monitor disease activity?

- ○ Yes
- ○ No
- ○ Only with new symptoms

17.Which imaging modality do you use most commonly to monitor disease activity?

- ○ None unless patient has symptoms
- ○ X ray
- ○ CT
- ○ Bone scan
- ○ MRI
- ○ PET-CT
- ○ Other

Please specify. ______________________

18.How soon do you repeat imaging to monitor treatment response after initiating immunosuppressant and/or bisphosphonate?

- ○ Never
- ○ 3 months
- ○ 6 months
- ○ 12 months
- ○ Other

____

19.Which MRI sequence do you think is helpful for evaluating CNO? CHOOSE ALL THAT APPLY.

- ☐ T1
- ☐ T2
- ☐ STIR or Fat Sat
- ☐ With contrast
- ☐ Other
- ☐ Unsure

Please specify. __________________________________

20.Which MRI findings do you think indicate active CNO disease? CHOOSE ALL THAT APPLY.

- ☐ Bone marrow edema
- ☐ Soft tissue inflammation
- ☐ Periosteal reaction
- ☐ Hyperostosis
- ☐ Joint effusion
- ☐ Synovium thickening
- ☐ Physeal irregularity, or bony bar
- ☐ Vertebral compression
- ☐ Fracture
- ☐ Other
- ☐ Unsure

Please specify. __________________________________

21.Which MRI findings in CNO are of most concern to you regarding poor long-term prognosis? CHOOSE ALL THAT APPLY.

- ☐ Bone marrow edema
- ☐ Soft tissue inflammation
- ☐ Periosteal reaction
- ☐ Hyperostosis
- ☐ Joint effusion
- ☐ Synovium thickening
- ☐ Physeal irregularity, or bony bar
- ☐ Vertebral compression
- ☐ Fracture
- ☐ Other
- ☐ Unsure

Please specify. __________________________________

22.Which of the following features define(s) active disease to you? CHOOSE ALL THAT APPLY.

- ☐ Patient complaining of pain localized to known sites
- ☐ Focal bone swelling and/or warmth
- ☐ Focal tenderness at known sites without allodynia
- ☐ Active arthritis
- ☐ Functional limitation of joints/limbs
- ☐ Elevated ESR and/or CRP
- ☐ Fever
- ☐ New lesions identified from imaging
- ☐ Abnormal MRI signals
- ☐ Other

Please specify __________________________________

23.In general, do you use an NSAID to initially treat CNO?

- ◯ Yes
- ◯ No

24.Which patients with active bone disease will you start DMARD, and/or biologics, and/or bisphosphonate if they do not have active IBD, arthritis or psoriasis? CHOOSE ALL THAT APPLY.

- ☐ Any patient regardless of disease duration or involved bone locations
- ☐ Refractory to NSAIDs treatment
- ☐ Long bone involvement with normal growth plate
- ☐ Vertebrae involvement with or without compression fracture
- ☐ Clavicle involvement
- ☐ Pelvic bone involvement
- ☐ Mandible involvement
- ☐ Cranial bone involvement
- ☐ Growth plate damage
- ☐ Pathological fracture
- ☐ Other, please specify

____

25.After your patient fails NSAIDs, which of these medications do you use as a second line treatment? CHOOSE ALL THAT APPLY.

- ☐ Methotrexate
- ☐ Sulfasalazine
- ☐ Other DMARD
- ☐ Glucocoticoid
- ☐ Pamidronate or other bisphosphonate
- ☐ TNF inhibitor
- ☐ Other

Please specify. ______________________________

26.How often do you prescribe glucocorticoids in patients experiencing a flare of disease?

- ◯ Never
- ◯ Rarely (< 25%)
- ◯ Sometimes (25-50%)
- ◯ Often (51-90%)
- ◯ Always (>90%)

27a. What is the daily dose (mg/kg) do you prescribe? ______________________________

27b. What is the max dose (mg/day) do you prescribe? ______________________________

28. How many weeks would you treat with glucocorticoid?

- ◯ 1-3 weeks
- ◯ 4-6 weeks
- ◯ 6-12 weeks
- ◯ >12 weeks

29.In general, how often do you use the following medications to treat CNO?

	Never	Rarely	Sometimes	Often	Always
Azithromycin	◯	◯	◯	◯	◯
Naproxen	◯	◯	◯	◯	◯
Celecoxib	◯	◯	◯	◯	◯
Indomethacin	◯	◯	◯	◯	◯
Other NSAIDs	◯	◯	◯	◯	◯
Methotrexate	◯	◯	◯	◯	◯
Sulfasalazine	◯	◯	◯	◯	◯
Other DMARD	◯	◯	◯	◯	◯
Glucocorticoid	◯	◯	◯	◯	◯
Etanercept	◯	◯	◯	◯	◯
Adalimumab or Infliximab	◯	◯	◯	◯	◯
Bisphosphonate	◯	◯	◯	◯	◯
Other	◯	◯	◯	◯	◯

Please specify. ______________________________

30.How long would you continue treatment before considering patients to have failed the treatment with no or limited response (i.e. persistent bone pain with warmth or swelling, elevated inflammation markers, active inflammation on imaging)?

	Never	1 month	2 months	3 months	4-6 months
NSAID	◯	◯	◯	◯	◯
DMARD	◯	◯	◯	◯	◯
BIOLOGIC	◯	◯	◯	◯	◯
GLUCOCORTICOID	◯	◯	◯	◯	◯
BISPHOSPHONATE	◯	◯	◯	◯	◯

31.Will you be interested in participating in a comparative effectiveness trial of different treatments in CNO?

◯ Yes
◯ No
◯ Unsure

32.Potential treatment arms for such a trial would include common treatments, alone or in combinations (NSAID, DMARD, Biologic, Bisphosphonate). Please indicate which treatment arms you would be interested in such a trial for newly diagnosed patients. CHOOSE ALL THAT APPLY.

☐ NSAID Alone
☐ DMARD Alone
☐ Biologic Alone
☐ Bisphosphonate Alone
☐ Biologic with DMARD
☐ Biologic with bisphosphonate
☐ Bisphosphonate with DMARD

33.In your opinion, which of the following criteria are required to define inactive disease in CNO? CHOOSE ALL THAT APPLY.

☐ Complete resolution of the pain at known CNO lesions
☐ Normal function of the affected sites
☐ Resolution of constitutional symptoms if present with active disease
☐ Absent focal tenderness/warmth/swelling of known CNO lesions on exam
☐ Resolution of synovitis if present with active disease
☐ Normal ESR, CRP
☐ No new CNO lesions confirmed by whole body MRI
☐ Absence of inflammation confirmed by imaging (if selected, specify which imaging tool?)
☐ Other, please specify

Please specify. ______________________________

34.How soon would you stop or taper medications after your patient achieves inactive disease?

◯ 1-3 months
◯ 4-6 months
◯ 7-12 months
◯ >12 months
◯ Depends on initial severity and/or location of affected sites.

Please specify your usual treatment strategy for each of the following 3 cases.

35.Case 1: a 12-year-old girl has a 3-month history of pain in her right arm. Her pain has progressively worsened so that she now has trouble moving her right shoulder. She denies weight loss or fever.
Imaging: An X ray shows a lytic lesion in proximal humerus and periosteal reaction around proximal third of humerus. A bone scan showed increased uptake of tracer in right humerus only.
Pathology: A bone biopsy showed neutrophils and plasma cells, negative for infection or malignancy.
Labs: Her CBC is normal. Acute phase reactants are mildly elevated (ESR 30 mm/hr, CRP 1.4 mg/dL).
Treatment: She was given Ibuprofen with mild improvement of her bone pain.
How would you treat this patient at this point? CHOOSE ALL THAT APPLY.

☐ Naproxen
☐ Indomethocin
☐ Other NSAID
☐ Methotrexate
☐ Sulfasalazine
☐ Other DMARD
☐ Etanercept
☐ Adalimumab or Infliximab
☐ Bisphosphonate
☐ Glucocorticoid
☐ Other

Please specify. __________________________________

36.If you choose bisphosphonate, which of following will you use?

◯ Pamidronate
◯ Zoledronic acid
◯ Other

Please specify. __________________________________

37.For your chosen bisphosphonate, what dose and frequency do you use? __________________________________

38.For your chosen bisphosphonate, how long will you treat this patient initially?

◯ 1 month
◯ 2 months
◯ 3 months
◯ 4-6 months
◯ >6 months

39.In the above case, how long would you continue treatment before stopping or tapering medications if there is a favorable response within 2-3 months (resolved pain, normalized ESR, CRP, improved imaging findings)?

◯ As soon as pain is resolved
◯ 2-3 months
◯ 4-6 months
◯ 7-12 months
◯ >12 months
◯ Other

Please specify. __________________________________

40.Case 2: a 5 year-old girl has had severe back pain for 2 months with nocturnal awakening. She complains of pain during the day and occasionally limps. She was initially seen by orthopedic surgeon who referred her to rheumatology.
Imaging: X ray of spine showed loss of height of T5. A spine MRI shows increased signal in vertebral bodies of T5 and T12 in STIR sequence.
Pathology: Bone biopsy from T5 vertebral body showed reactive bone formation.
Labs: Her blood culture, tissue culture, and PPD were negative. CBC was normal. ESR was 20 mm/hr and CRP was 0.5 mg/dL.
Treatment: She was begun on Naproxen two weeks ago which improved her back pain slightly. Her night pain and limping resolved.
How would you treat this patient at this point? CHOOSE ALL THAT APPLY.

☐ Continue Naproxen
☐ Switch from Naproxen to Indomethocin
☐ Switch from Naproxen to other NSAID
☐ Methotrexate
☐ Sulfasalazine
☐ Other DMARD
☐ Etanercept
☐ Adalimumab or Infliximab
☐ Bisphosphonate
☐ Glucocorticoid
☐ Other

Please specify. __________________________________

41.If you choose bisphosphonate, which of following will you use?

- ◯ Pamidronate
- ◯ Zoledronic acid
- ◯ Other

Please specify. ______________________________

42.For your chosen bisphosphonate, what dose and frequency do you use? ______________________________

43.For your chosen bisphosphonate, how long will you treat this patient initially?

- ◯ 1 month
- ◯ 2 months
- ◯ 3 months
- ◯ 4-6 months
- ◯ >6 months

44.In the above case, how long would you continue treatment before stopping or tapering medications if there is a favorable response within 2-3 months (resolved pain, normalized ESR, CRP, improved imaging findings)?

- ◯ As soon as pain is resolved
- ◯ 2-3 months
- ◯ 4-6 months
- ◯ 7-12 months
- ◯ >12 months
- ◯ Other

Please specify. ______________________________

45.Case 3: a 15-year-old boy has had right hip pain with intermittent limping for the past year. He has had slight improvement in symptoms with manipulation by a chiropractor. His pain worsened.
Imaging: Initial X rays showed normal hips and SI joints. An MRI revealed multiple sites (sacrum ala, acetabulum and ischia) in his pelvis with increased signal in STIR sequence.
Pathology: A bone marrow biopsy showed normal tri-lineages and focal fibrosis, and bone biopsy from sacral ala revealed fragments of devitalized bone.
Labs: Bacterial and AFB cultures from bone biopsy were negative.
Treatment: He was treated with naproxen and with initial quick improvement.
Followup: At his 2 months follow up appointment, he still complains of pain in his right hip. A repeat MRI showed persistent marrow edema in the above sites but the soft tissue inflammation has improved.
How would you treat this patient at this point? CHOOSE ALL THAT APPLY.

- ☐ Continue Naproxen
- ☐ Switch from Naproxen to Indomethocin
- ☐ Switch from Naproxen to other NSAID
- ☐ Methotrexate
- ☐ Sulfasalazine
- ☐ Other DMARD
- ☐ Etanercept
- ☐ Adalimumab or Infliximab
- ☐ Bisphosphonate
- ☐ Glucocorticoid
- ☐ Other

Please specify. ______________________________

46.If you choose bisphosphonate, which of following will you use?

- ◯ Pamidronate
- ◯ Zoledronic acid
- ◯ Other

Please specify. ______________________________

47.For your chosen bisphosphonate, what dose and frequency do you use? ______________________________

48.For your chosen bisphosphonate, how long will you treat this patient initially?

- ◯ 1 month
- ◯ 2 months
- ◯ 3 months
- ◯ 4-6 months
- ◯ >6 months

49.In the above case, how long would you continue treatment before stopping or tapering medications if there is a favorable response within 2-3 months (resolved pain, normalized ESR, CRP, improved imaging findings)?

◯ As soon as pain is resolved
◯ 2-3 months
◯ 4-6 months
◯ 7-12 months
◯ >12 months
◯ Other

Please specify. __________________________________

50. CRMO/CNO research group currently aim to develop consensus treatment plans and set up a longitudinal registry and repository study. What other research areas would you recommend further development?
